# Supplementary material for: Dynamic transcriptomic profiles of zebrafish gills in response to zinc supplementation
Source: BMC Genomics. 2010 Oct 11;11:553. doi: 10.1186/1471-2164-11-553 (PMC3091702; doi:10.1186/1471-2164-11-553)
Supplement: Additional file 2 — Interactive Direct Interaction Network representing the molecular interactions between zinc, copper, iron, calcium and proteins encoded by transcripts changed by zinc supplementation. Mini web-site containing index.html and hyperlinked pages in subdirectory describing a Direct Interaction Network automatically generated based on curated interactions contained within the proprietary PathwayArchitect database. Ovals represent proteins and the circles symbolize metal ions. Objects are coloured by their abundance in zebrafish at the time-point they were significantly different from the control is a scale from -4 fold (dark green) to +4 fold (dark red). Where significant differences were found at more than one time-point, the colour overlay shows expression at the first instance. Dark blue squares denote 'binding', and light blue squares 'expression'; green squares stand for 'regulation', green diamonds for 'metabolism', and green circles for 'promoter binding'. Arrow heads indicate directionality of the interaction where annotated. All nodes and edges can be further interrogated by selecting the relative area of the image. [file 1471-2164-11-553-S2.zip › PathwayArchitect Zn xs DIN/672760.html]

# REGULATION:

|  |  |
| --- | --- |
| Type | REGULATION |
| Effect | None |


---

|  |  |
| --- | --- |
| Score | 0 |


---

|  |  |
| --- | --- |
| Reference Count | 3 |


---

|  |  |
| --- | --- |
| Mechanism | Unknown |


---

|  |  |
| --- | --- |
| Reference:0 || Sentence | "In single HEK-293 cells, ATP and carbamylcholine (CCh) stimulated Ca(2+) release from intracellular stores via a pathway that was entirely dependent on Ins(1,4,5)P(3)." |
| PMID | 11042123 |
| Year | 2000 |
| Species | Human |
| Journal | Biochem J |
| RefScore | 0 |
| Source | PArchNLP |
  |
|  |  |


---

|  |  |
| --- | --- |
 Reference:1 || Sentence | "For example, in permeabilized L1210 mouse lymphoma cells, 10-50 microM Al3+ activated Ins(1,3,4,5)P4-dependent Ca2+ mobilization and Ins(1,3,4,5)P4 3-phosphatase activity [Loomis-Husselbee, Cullen, Irvine and Dawson (1991) Biochem." |
| PMID | 7832774 |
| Year | 1995 |
| Species | Human |
| Journal | Biochem J |
| RefScore | 1 |
| Source | PArchNLP |
  ||  |  |


---

|  |  |
| --- | --- |
 Reference:2 || Sentence | "The present findings show that agonist-stimulation significantly increased the levels of Ins(1,4,5)P3 and Ins(1,3,4,5)P4 after only 5 s, indicating that one or both of these substances could play a role in the regulation of Ca2+ influx." |
| PMID | 2163689 |
| Year | 1990 |
| Species | Human |
| Journal | Biochim Biophys Acta |
| RefScore | 1 |
| Source | PArchNLP |
  |


---

|  |  |
| --- | --- |
